# Supplementation of *Ampelopsis grossedentata* extract contributes to the improvement of intestinal health in swine

**DOI:** 10.3389/fvets.2024.1417309

**Published:** 2024-08-21

**Authors:** Xiangyan Liu, Fusheng Zhang, Mengyao Li, Rong Li, Zhen Zhang, Juan Xu, Lixin Wen, Rongfang Li

**Affiliations:** ^1^Hunan Engineering Research Center of Livestock and Poultry Health Care, College of Veterinary Medicine, Hunan Agricultural University, Changsha, China; ^2^Changsha Lvye Biotechnology Co., Ltd., Changsha, China

**Keywords:** *Ampelopsis grossedentata*, antioxidant ability, inflammation, gut microbiota, pig, intestinal immunity

## Abstract

**Introduction:**

*Ampelopsis grossedentata* (vine tea), a high polyphenol content antioxidant plant resource, is renowned for its medicinal benefits. This study aimed to investigate the effects of *Ampelopsis grossedentata* extract (AGE) on anti-inflammatory and antioxidant ability, enhancement of intestinal immunity, improvement of intestinal structure, and regulation of gut microbiota in swine.

**Methods:**

A total of 135 weaned piglets were randomly divided into three groups: a control group, a low-dose group, and a high-dose group. Pigs were weighed and blood was collected on days 36, 85, and 154. The feed intake was recorded daily to calculate growth performance parameters. On day 154, five to six pigs in each group were randomly selected and euthanized to obtain a small intestine to investigate the effects of AGE on anti-inflammatory and antioxidant abilities and gut microbiota.

**Results:**

The results showed that 500 mg/kg AGE increased the expression of anti-inflammatory and immune cytokines (IL-10, IgG, and IgA) (*p* < 0.05, *p* < 0.01) and decreased the expression of proinflammatory cytokines (IL-1β) (*p* < 0.05) in serum. Additionally, 500 mg/kg AGE enhanced the antioxidant capacity by increasing the GSH-Px, CAT, and SOD (*p* < 0.05, *p* < 0.01).

**Discussion:**

A total of 500 mg/kg AGE significantly increased the abundance of gut microbiota, enhanced the gut barrier, and modulated gut immunity. During the piglet phase, 500 mg/kg AGE increased the relative abundance of *Prevotella* (*p* < 0.05). During the growing-finishing phase, 500 mg/kg AGE increased the relative abundance of *unclassified_f__Lachnospiraceae* and *Bacteroides* (*p* < 0.05, *p* < 0.01). Overall, we recommended 500 mg/kg AGE as a routine addition dose for swine to improve porcine growth performance and intestinal health.

## 1 Introduction

*Ampelopsis grossedentata* (vine tea), a new food resource in recent years, is widely used to treat common health problems and promote animal production (1, 2). Vine tea has many biological functions because it contains rich active ingredients, including flavonoids, polysaccharides, alkaloids, and some polyphenols (3). It has been reported that *Ampelopsis grossedentata* has a variety of pharmacological properties, such as anti-oxidation, anti-thrombosis, anti-inflammatory activity, antitumor effect, and cardiovascular protection (4–7). Additionally, plant-derived polyphenols exhibit potent antioxidant properties, enabling them to scavenge free radicals and alleviate disorders associated with oxidative stress (8, 9). The main function of the intestine is to digest and absorb nutrients (10), and it is the major organ targeted by free radicals (11). Hence, the antioxidant function of the intestine is very important and closely related to the pig production industry. Improving the oxidative stress response of the intestine has become a paramount concern in the swine industry.

As is known to all, trillions of microbes inhabit the intestinal tract of mammals, where they play extensive biological roles in animals’ health (12). In the aspect of the feed efficiency of swine, mounting evidence has indicated that the gut microbiota plays a pivotal role in the absorption of nutrients, energy, and carbohydrate metabolism (13). Furthermore, recent research has demonstrated that the average daily gain, body weight, and nutrient digestibility of pigs are related to the composition, structure, and abundance of their gut microbiota (14, 15). Despite the fact that previous studies have demonstrated the ability of dihydromyricetin (DMY), the primary medicinal substance of *Ampelopsis grossedentata*, to modulate the composition and interactions of gut microbiota in rats (16), the influence and function of AGE on gut microbiota in pigs at different periods are still unclear.

Therefore, in this study, we aimed to investigate the impact of AGE on growth performance, inflammatory factors, antioxidant ability, reflection of tight junction proteins in different segments of the small intestine, and diversity of colonic microbiota in pigs. Different doses of AGE were administered to explore its potential effects on inflammation and the gut microbiota and its potential protective benefits for the gut.

## 2 Materials and methods

### 2.1 Ethics statement

All experimental procedures in this study were reviewed and approved by the Animal Care and Use Committee of Hunan Agricultural University (located in Changsha, Hunan Province, China) (No.2022093).

### 2.2 Animal management and experimental procedures

A total of 135 male Duroc × Landrace × Yorkshire piglets (weaned at 21 d of age; the weight reached 10.13 ± 0.18 kg after 10 days of adaptive feeding with basal diet) were assigned to three groups at random: a control group (fed with basal diet), a low-dose group (fed with a basal diet supplemented with AGE) and a high-dose group (fed with basal diet supplemented with AGE). The experimental period of animals was 154 days, fed with 500 mg/kg AGE on the 0–85th day and 250 mg/kg AGE on the 85th–154th day and a high-dose group fed with 1,000 mg/kg AGE on the 0–85th day and 500 mg/kg AGE on the 85th–154th day. Due to the large amount of feed intake during the pig stage and the existence of some physiological conditions, the dosage of additives is usually reduced by half to achieve the expected effect. Each group consisted of 45 piglets, with three replicates. Blood samples were collected on the 36th, 85th, and 154th day of the experiment, and growth performance data were recorded. Serum oxidation indicators and inflammatory factors were detected. On day 154, five to six pigs in each group were randomly selected and humanely slaughtered. Subsequently, each intestine was immediately frozen in liquid nitrogen and stored at −80°C for further analysis. The AGE used in the experiment was purchased from Yongzhou Huamao Biotechnology Co., Ltd., and the content of DMY extract is 20.15%, which is detected by the Ch.P.C.Rule 23-HPLC test method. The basal diet formulas are shown in Table 1. The nutrient content is entrusted to the Institute of Subtropical Agroecology, Chinese Academy of Sciences for testing (17).

**Table 1:** Ingredient and nutrient composition of basal diets.

Item	Period		
	Weaning(0–36d)	Growing(36–85d)	Finishing(85–154d)
Ingredient, % as-fed			
Corn	54.44	45.95	45.8
Rice	10.08	18.18	22.39
Wheat		2.02	
Soybean meal, 46%	18.15		
Soybean meal, 43%		23.23	20.35
Rice bran oil		5.05	6.11
Puffed soybean	3.02		
Fermented soybean meal	1.81		
Compound fermentation material	2.02		
Soybean oil	0.5	0.4	
Inferior flour	2.02	2.02	3.05
Total tryptophan	0.02		
Total threonine	0.13	0.02	
Total methionine	0.1	0.02	
Lysine salt	0.5	0.27	0.24
Choline chloride		0.08	0.08
Rice bran meal	0.3		
Rice hulls		0.61	
Calcium hydrogen phosphate	1.25	0.74	0.51
Rock powder	0.6		1.06
Sodium chloride	0.3	1.01	0.41
White sugar	1.84	0.4	
Acidifier	0.5		
Fish meal	2.42		
Total	100	100	100
Nutritional level detection value, %			
Asp	1.59	1.65	1.39
Thr	0.65	0.78	0.58
Ser	0.78	0.86	0.69
Glu	2.88	2.97	2.55
Gly	0.69	0.77	0.62
Ala	0.87	0.92	0.77
Val	0.79	0.83	0.71
Met	0.00	0.01	0.00
Ile	0.67	0.70	0.60
Leu	1.43	1.48	1.25
Tyr	0.45	0.47	0.37
Phe	0.79	0.81	0.70
Lys	0.98	1.24	0.84
His	0.41	0.42	0.36
Arg	0.99	1.00	0.86
Pro	0.96	1.04	0.86
Ca	0.7	0.671	0.571
CP	16.6	18.3	15.1
CFat(g/100 g)	3.6	3.9	3.5
P	0.384	0.52	0.432
*CF*	4.1	3.2	4.3

Based on the requirement of the National Research Council (NRC, 2012), the diet was formulated primarily by ground corn–soybean basal meal without any antibiotics. Asp, Aspartic acid; Thr, threonine; Ser, serine; Glu, glutamine; Gly, glycine; Ala, alanine; Val, valine; Met, methionine; Ile, isoleucine; Leu, leucine; Tyr, tyrosine; Phe, phenylalanine; Lys, lysine; His, histidine; Arg, arginine; Pro, proline; Ca, calcium; P, phosphorus; CP, crude protein; CFat, crude fat; CF, crude fiber.

### 2.3 Histological evaluation of the small intestine

For histological evaluation, the fixed segments of the small intestine were initially dehydrated using ethanol and isopropanol, followed by embedding in paraffin. The specimens were then sectioned into slides and stained with hematoxylin and eosin (H&E) for analysis.

### 2.4 Measurement of antioxidative capacity

The serum and small intestine tissues were used to measure the antioxidant capacity. The levels of superoxide dismutase (SOD), malondialdehyde (MDA), catalase (CAT), and glutathione peroxidase (GSH-Px) were determined using colorimetric tests, and assay kits obtained from Nanjing Jiancheng Bioengineering Institute Co., Ltd., Nanjing, China.

### 2.5 Measurement of inflammatory cytokines and immune factors

The levels of TNF-α, IL-4, IL-1β, IL-10, IgG, and IgA in serum were assessed using ELISA kits (Jiangsu Meimian Industrial Co., Ltd., Jiangsu, China) according to the manufacturer’s instructions. The concentrations of sIgG and sIgA in small intestine samples were determined using assay kits (Jiangsu Jingmei Biological Technology Co., Ltd., Jiangsu, China).

### 2.6 RNA extraction and real-time quantitative PCR

Total RNA was extracted separately from the duodenum, jejunum, and colon using TRIzol (Hunan Akerui Bioengineering Co., Ltd., Changsha, China). Quantitative PCR (qPCR) was performed using a real-time PCR system (StepOne, Foster, CA, USA) based on the mRNA sequences of porcine *IL-10*, *IL-1β*, *zo-1*, *claudin-1*, *occludin*, and *β-actin* in GenBank. The RT-qPCR reaction system and conditions were carried out according to the instructions provided with the Escrow fluorescent qPCR kit (Hunan Akerui Bioengineering Co., Ltd., Changsha, China). The calculated value using the 2^−△△Ct^ method was used to analyze data. The primers utilized are listed in Supplementary Table S1.

### 2.7 Western blotting analysis

An equal amount of protein (50 μg) from each duodenal, jejunal, and ileum sample was separated by electrophoresis on SDS-PAGE along with prestained protein markers. According to the molecular weight of the target protein, 10–15% separation gel was prepared and the concentration of the gel was 5%. The concentrated glue had a constant pressure of 60 V for approximately 30 min. The separation gel was kept at a constant pressure of 120 V, and the electrophoresis stop time was determined by a pre-dyeing protein marker. Then it was transferred to PVDF membranes using the wet transfer method. After blocking with gelatin at room temperature for 4 h, membranes were subsequently incubated with primary antibodies of β-Actin (Proteintech, 20,536-1-AP), Occludin (AiFang, AF300990), Claudin-1 (Abmart, T56872), and ZO-1 (Affinity Biosciences, AF5145) at a dilution of 1:1,000 overnight at 4°C. After three times washing and then incubation with the secondary antibody (Thermo Fisher Scientific, 31,460) at a dilution of 1:10,000 for 45 min at room temperature. Then, the blots were exposed to an ECL detection reagent (Yeasen, 36208ES60) for 1 min to detect chemiluminescence signals and visualized using BIO-RAD Universal Hood II (Bio-Rad Laboratories, Hercules, USA). Densitometry was performed using ImageJ software (National Institutes of Health, Bethesda, USA).

### 2.8 16S rRNA gut microbiota analysis

The total bacterial DNA was extracted from colonic content samples (*n* = 6 piglets per group, *n* = 5 growing-finishing pigs per group) using the HiPure Stool DNA kits (Magen, Shanghai, China) according to the manufacturer’s instructions. The V3–V4 regions of the 16S rRNA were amplified using the universal primers 338F and 806R. Paired-end sequenced on an Illumina MiSeq PE300 platform/NovaSeq PE250 platform (Illumina, San Diego, CA, USA) following standard protocols. Raw data obtained from gut microbiota were processed using the Majorbio I-Sanger Cloud Platform.^1^ The sequences were then subjected to OTU clustering using UPARSE software, with a similarity threshold of 97%. Each sequence was annotated by species classification using RDP Classic and compared to the Silva 138 database with a confidence threshold of 0.7. The effects of AGE on the gut microbiota of swine were compared through the analysis of OTU, which included analyzing alpha diversity, beta diversity, species composition analysis, LEfSE, and BugBase difference analysis. More specific details of the microbial sequencing process were described by Qu et al. (18).

### 2.9 Statistical analysis

IBM SPSS Statistics 25.0 (IBM Corp, Armonk, NY, United States) software was used to analyze the data. The full factorial model was used to analyze the results of animal production performance, including the fixed effect of treatment (Trt), time (Time), and the interaction between treatment and time (Trt × Time), while each animal was taken as a random factor. All results are shown as the means ± standard error (x̄ ± SEM). Data related to inflammation, antioxidant activity, immunoglobulin (Ig) contents, intestinal barrier, and gut microbiota were assessed through one-way parametric analysis of variance (ANOVA), followed by Tukey’s *post-hoc* test according to the homogeneity of variance. If the variances were not neat, the one-way non-parametric ANOVA (Kruskal–Wallis test) followed by Duncan’s tests was used. In all analyses, the *p*-values of <0.05 were considered indicative of a significant difference, while *p*-values of <0.01 were considered indicative of an extremely significant difference. GraphPad Prism 9 software was used for graphic processing.

## 3 Results

### 3.1 Effects of age on the growth properties of piglets and growing-finishing pigs

The body weight of growing-finishing pigs increased with age (Table 2). The addition of a high dose of AGE to the diet had no significant effect on the average daily weight gain (ADG) and the average daily feed intake (ADFI) of pigs (*p* > 0.05) but significantly reduced the feed conversion ratio (FCR) (*p* < 0.05) throughout the entire experimental period.

**Table 2:** Growth performance of piglets and growing-finishing pigs.

Item	treatment groups				*P*-value		
	Con	Low	High	SEM±	Trt	Time	Trt × Time
BW^1^, kg					0.737	<0.001	0.741
0 d	10.13	10.32	10.11	0.14			
36 d	21.94	23.23	22.89	0.31			
85 d	61.04	59.76	59.26	0.70			
154 d	132.91	130.25	131.50	1.07			
ADG^2^, g/d					0.550	<0.001	0.288
0–36 d	327.90	358.78	354.94	0.01			
36–85 d	728.71	687.07	685.16	0.01			
85–154 d	1041.47	1021.46	1058.18	0.01			
0–154 d	775.14	760.15	775.10	0.01			
ADFI^3^, kg/d					0.048	<0.001	0.866
0–36 d	0.58	0.57	0.57	0.01			
36–85 d	1.64	1.61	1.58	0.02			
85–154 d	3.25	3.17	3.13	0.03			
0–154 d	2.11	2.07	2.04	0.01			
FCR^4^, kg/ kg					0.004	<0.001	0.021
0–36 d	1.76^a^	1.62^b^	1.60^b^	0.03			
36–85 d	2.25	2.34	2.31	0.05			
85–154 d	3.12^a^	3.10^ab^	2.96^b^	0.03			
0–154 d	2.52^a^	2.51^ab^	2.44^b^	0.02			

^a, b^Data are expressed as the x̄ ± SEM (*n* = 135). Different superscript letters in the same row indicate significant differences (*p* < 0.05). 1 BW = body weight. 2 ADG = average daily gain. 3 ADFI = average daily feed intake. 4 FCR = feed intake to body gain ratio.

### 3.2 Antioxidant effect of age on serum and intestinal levels of piglets and growing-finishing pigs

**Figure 1:**
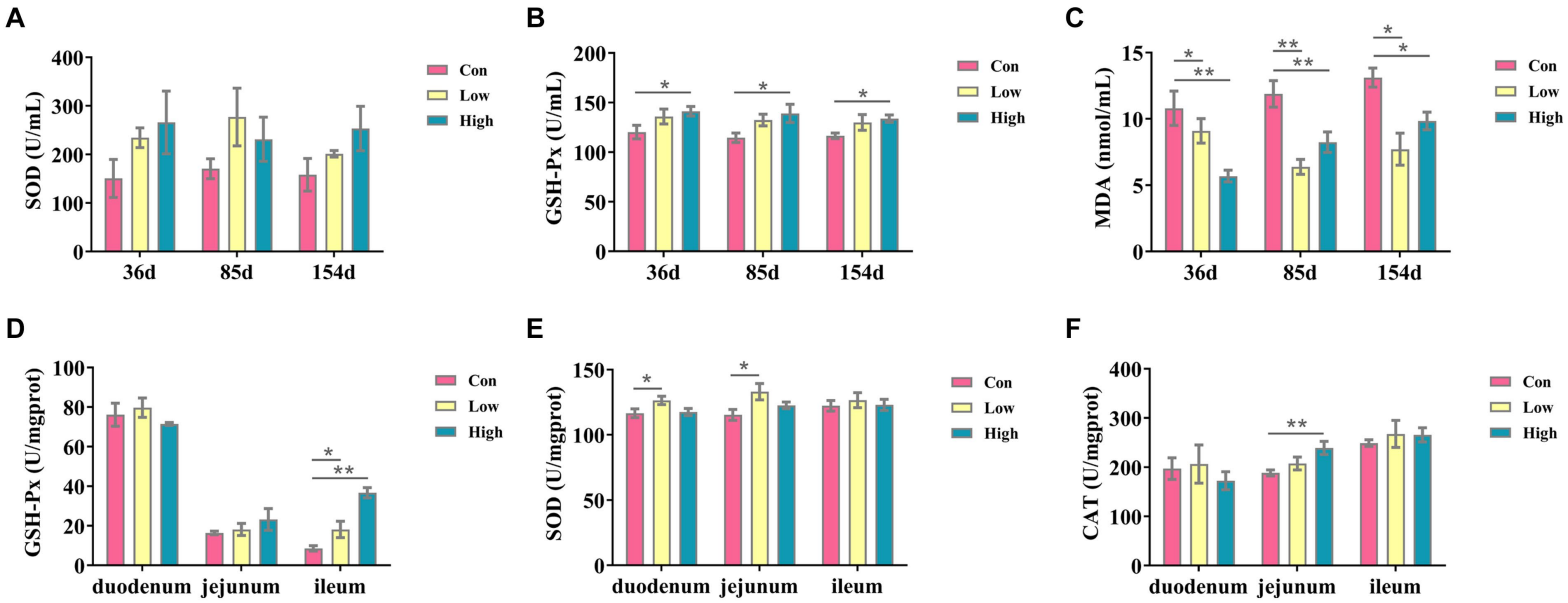
Antioxidant factors in serum and small intestine. The antioxidants in serum include the following: **(A)** SOD; **(B)** GSH-Px; **(C)** MDA; the antioxidant indicates in intestine tissues, including the following: **(D)** GSH-Px; **(E)** SOD; and **(F)** CAT. Data are expressed as the x̄ ± SEM (*n* = 5). Statistical significance compared with the control, *p < 0.05 indicates significant differences, whereas **p < 0.01 indicates extremely significant differences.

The antioxidant indicators are presented in Figure 1. Compared with the control group, the serum SOD and GSH-Px levels were increased, and MDA level was decreased in the low-dose and high-dose groups during the three periods, with significant differences between GSH-Px and MDA (*p* < 0.05, *p* < 0.01, Figures 1B,C). Moreover, in the low-dose groups, antioxidant indices of GSH-Px activity in the ileum and SOD activity in the duodenum and jejunum were significantly increased (*p* < 0.05, Figures 1D,E). The GSH-Px in the ileum and CAT in the jejunum were significantly increased in the high-dose groups (*p* < 0.01, Figures 1D,F). These results suggested that AGE improved antioxidant ability in piglets and growing-finishing pigs.

### 3.3 Effect of age on serum inflammatory factor and immunoglobulin content of piglets and growing-finishing pigs

**Figure 2:**
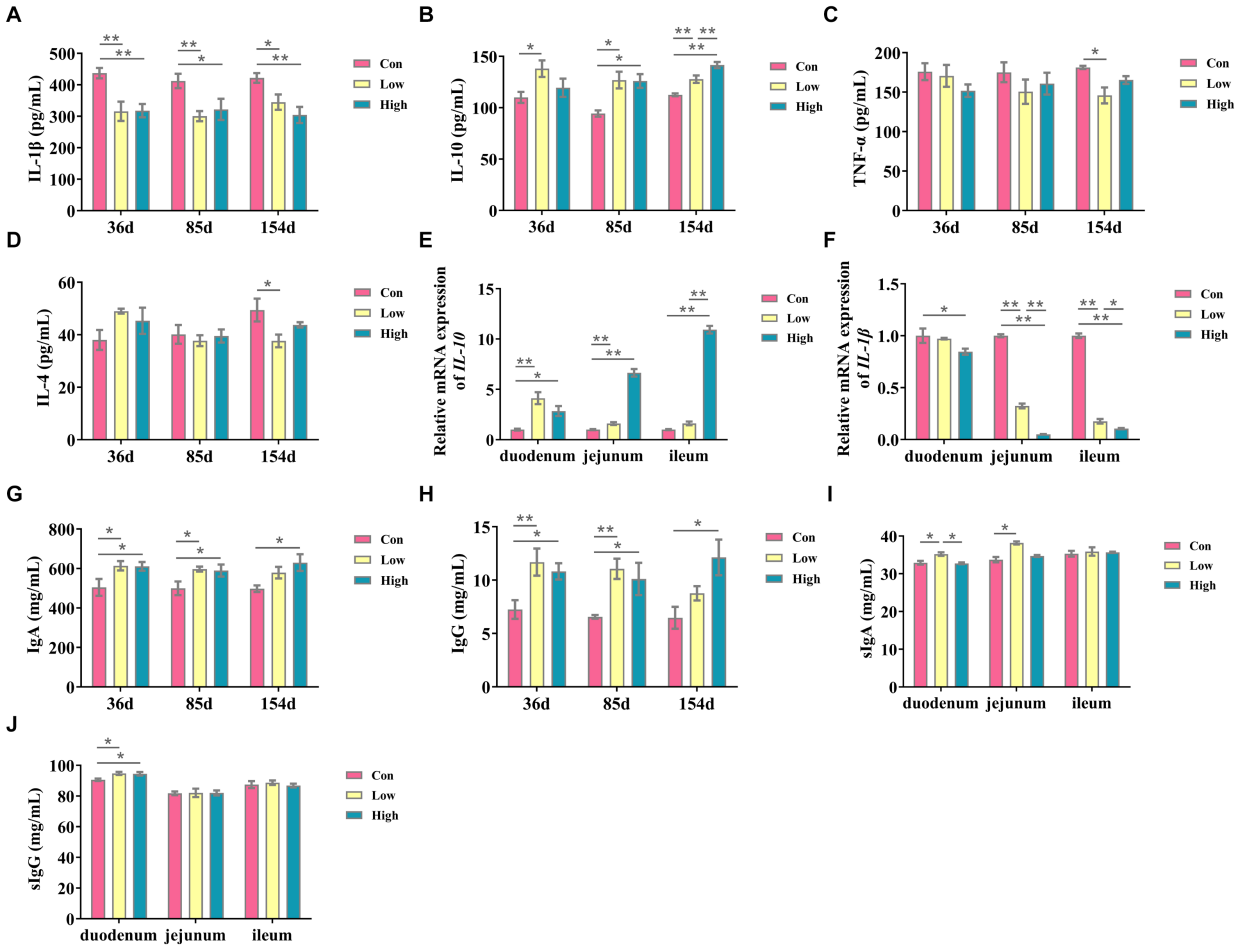
Inflammatory cytokines and immunoglobulin contents in serum and small intestine. **(A)** IL-1β; **(B)** IL-10; **(C)** IL-4; and **(D)** TNF-*α*; **(E)** the mRNA expressions of *IL-10*; **(F)** the mRNA expressions of *IL-1β;* **(G)** Ig A, immunoglobulins A; **(H)** Ig G, immunoglobulins G; **(I)** slgA, secretory immunoglobulin A; and **(J)** slgG, secretory immunoglobulin G. Data are expressed as the x̄ ± SEM (*n* = 5). Statistical significance compared with the control, *p < 0.05 indicates significant differences, whereas **p < 0.05 indicates extremely significant differences.

As depicted in Figure 2, the experimental groups exhibited a significant reduction in the inflammatory factor IL-1β when compared to the control group (*p* < 0.05, *p* < 0.01, Figure 2A). Conversely, there was a substantial increase in the anti-inflammatory factor IL-10 (*p* < 0.05, *p* < 0.01, Figure 2B) throughout the entire duration of the experiment. Similarly, the serum concentrations of Ig, including IgA and IgG, were significantly increased (*p* < 0.05, *p* < 0.01, Figures 2G,H). After 154 days of experimentation, the mRNA expression levels of *IL-10* in the small intestine were significantly elevated (*p* < 0.05, *p* < 0.01, Figure 2E), while *IL-1β* exhibited a significant decrease (*p* < 0.05, *p* < 0.01, Figure 2F) when compared to the control group. In the low-dose group, the concentrations of secretory immunoglobulin A (sIgA) in the duodenum and jejunum were significantly increased (*p* < 0.05, Figure 2I), and the concentrations of secretory immunoglobulin G (sIgG) in the duodenum were significantly increased in both the low-dose group and high-dose group (*p* < 0.05, Figure 2J). These findings suggest that AGE enhances anti-inflammatory activity and improves intestinal immunity in piglets and growing-finishing pigs.

### 3.4 Protective effects of age on the intestinal tract of growing-finishing pigs

**Figure 3:**
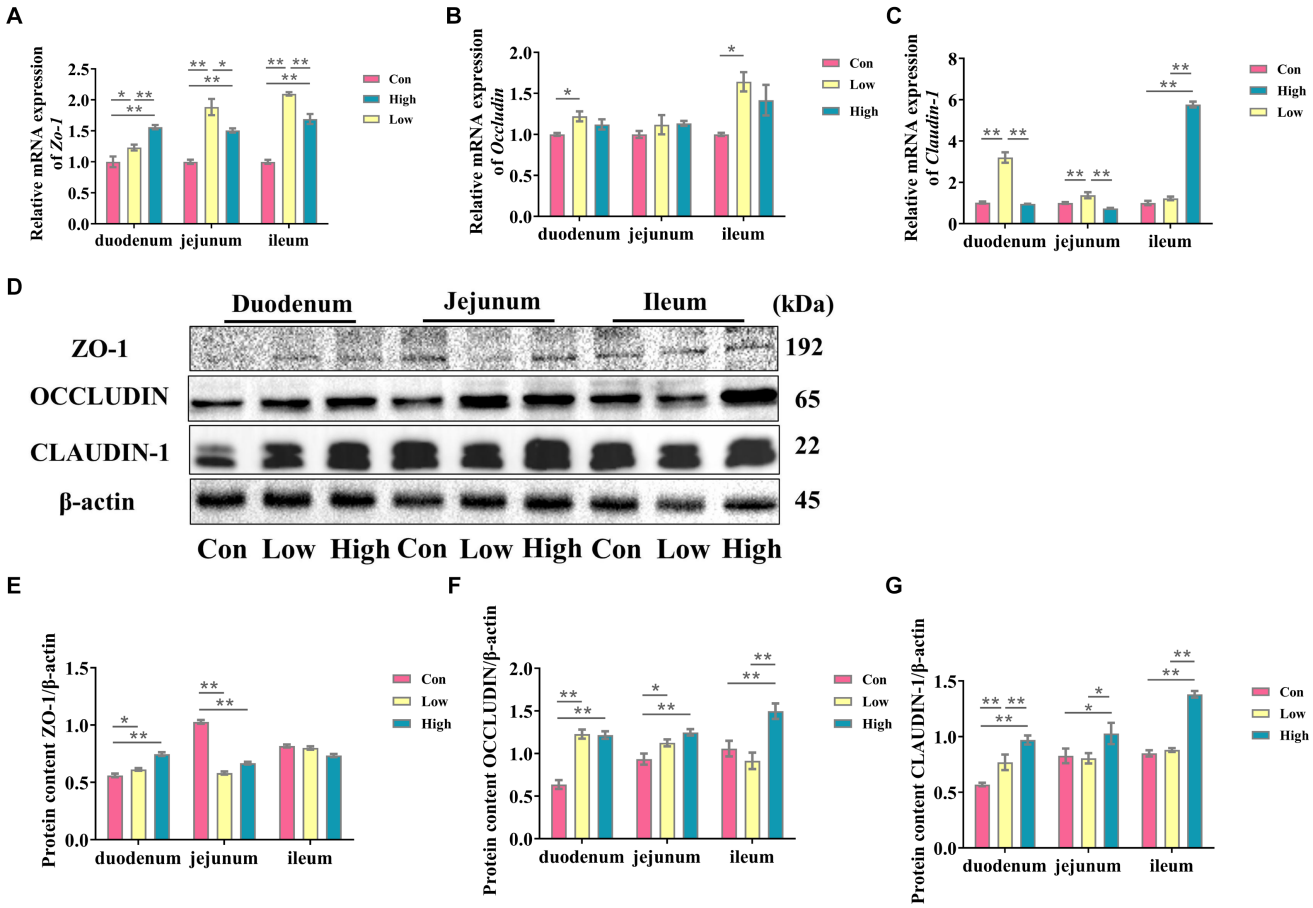
AGE is beneficial for improving the intestinal barrier of weaned piglets. **(A–C)** The mRNA expressions of *Zo-1*, *Claudin-1*, *and Occludin* in the duodenum, jejunum, and ileum; **(D)** the representative bands for the western blot of ZO -1, OCCLUDIIN, and CLAUDIN-1; **(E–G)** The levels of ZO-1/β-actin, OCCLUDIIN /β-actin, and CLAUDIN-1/β-actin in the duodenum, jejunum, and ileum. Data are expressed as the x̄ ± SEM (*n* = 5). Statistical significance compared with the control, *p < 0.05 indicates significant differences, whereas **p < 0.01 indicates extremely significant differences.

The intestinal morphology and barrier are shown in Table 3 and Figure 3. In comparison to the control group, both the low-dose and high-dose groups increased the mRNA expression levels of *Zo-1* in the small intestine (*p* < 0.05, *p* < 0.01, Figure 3A). Furthermore, the low-dose group demonstrated a significant increase in the mRNA expression level of *occludin* in the duodenum and ileum (*p* < 0.05, Figure 3B). While the low-dose group exhibited a significant increase in the mRNA expression level of *claudin-1* in the duodenum and jejunum, the high-dose group showed a significant increase in the ileum (*p* < 0.01, Figure 3C). Additionally, the protein expression levels of ZO-1, OCCLUDIN, and CLAUDIN-1 in the small intestine were significantly increased in the high-dose group (*p* < 0.05, *p* < 0.01, Figures 3E–G), except for the protein expression level of ZO-1 in the ileum. In terms of intestinal morphology, the high-dose group exhibited enhanced small intestine villus height and villus height to crypt depth ratio compared to the control group (Table 3). These results suggest that AGE improves intestinal morphology and intestinal barrier function in growing-finishing pigs.

**Table 3:** Effects of vine tea extract on the intestinal morphology of pigs.

Item	Con	Low	High	SEM±	*P*-value	Linear	Quadratic
Duodenum							
Villus height, μm	302.67^b^	322.72^b^	381.38^a^	13.22	0.029	0.010	0.029
Crypt depth, μm	133.73	132.52	125.13	3.65	0.610	0.351	0.610
Villus height: crypt depth ratio	2.28^b^	2.44^b^	3.06^a^	0.10	<0.001	<0.001	<0.001
Jejunum							
Villus height, μm	360.43^b^	424.30^a^	449.98^a^	11.67	<0.001	<0.001	<0.001
Crypt depth, μm	219.22^ab^	207.28^a^	285.88^b^	5.66	0.040	0.011	0.040
Villus height: crypt depth ratio	1.84^b^	2.05^b^	2.43^a^	0.08	0.004	<0.001	0.004
Ileum							
Villus height, μm	265.15^b^	278.65^b^	331.70^a^	7.96	<0.001	<0.001	<0.001
Crypt depth, μm	131.35^b^	125.98^ab^	109.65^a^	4.44	0.111	0.042	0.111
Villus height: crypt depth ratio	2.05^b^	2.22^b^	3.07^a^	0.13	<0.001	<0.001	<0.001

Different superscript letters (a, b, and c) indicate significant differences (*P* < 0.05).

### 3.5 Dietary age supplementation shifted gut microbial diversity in piglets and growing-finishing pigs

**Figure 4:**
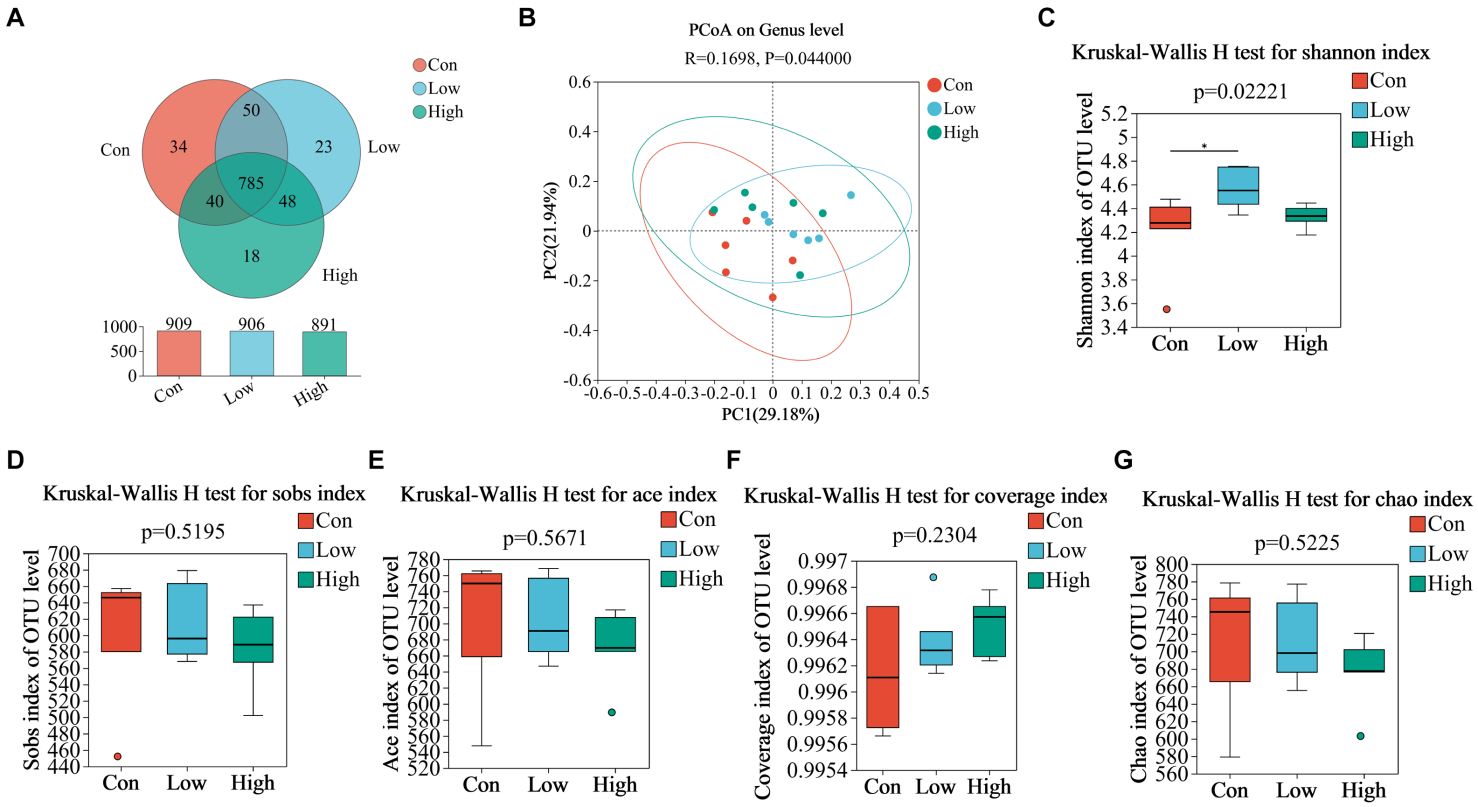
**(A)** Venn diagram of OTU distribution among groups of piglets; **(B)** PCoA based on weighted Bray–Curtis distance. PERMANOVA was used to detect the significance of separation between groups; **(C–G)** Alpha diversity of intestinal microbial piglets in different groups. Data are expressed as the x̄ ± SEM (*n* = 6). Statistical significance compared with the control, *p < 0.05 indicates significant differences.

**Figure 5:**
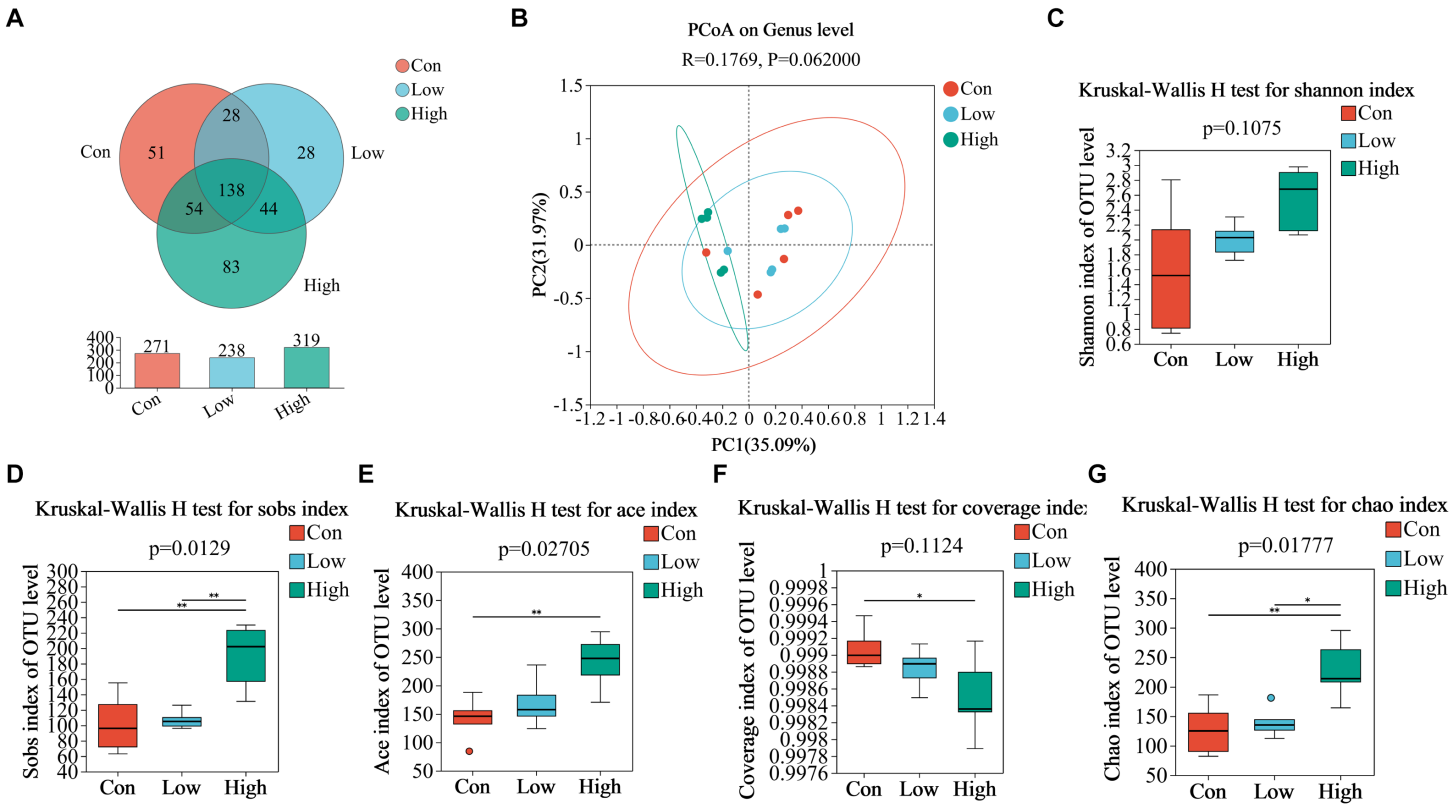
**(A)** Venn diagram of OTU distribution among groups of pigs; **(B)** PCoA based on weighted Bray–Curtis distance. PERMANOVA was used to detect the significance of separation among groups; **(C–G)** alpha diversity of intestinal microbial of pigs in different groups. Data are expressed as the x̄ ± SEM (*n* = 5). Statistical significance compared with the control, *p < 0.05 indicates significant differences, whereas **p < 0.01 indicates extremely significant differences.

We analyzed fecal microbiota through high-throughput sequencing of 16Sr RNA amplicons. The Venn diagram demonstrated that there were 785 common OTUs across all three groups during the piglet phase, with 34, 23, and 18 unique OTUs in the con, low, and high groups, respectively (Figure 4A). In the growing-finishing pig phase, there were 136 common OTUs in all three groups, with 51, 28, and 83 unique OTUs in the con, low, and high groups, respectively (Figure 5A). PCoA analysis revealed significant differences in the gut microbiota composition among the groups (Figure 4B, *P* = 0.044 by PERMANOVA, Figure 5B, *P* = 0.062 by PERMANOVA.). Alpha diversity analysis showed as Figures 4C–G, 5C–G, the low-dose group exhibited an increase in the Shannon index during the piglet phase (*p* < 0.05, Figure 4C). In the growing-finishing pig phase, the high-dose group showed an increase in the Sobs, Ace, and Chao indices (*p <* 0.05, *p* < 0.01, Figures 5D,E,G). These results suggest that AGE treatments induce alterations in the structure and diversity of gut microbiota in both piglets and growing-finishing pigs.

### 3.6 Dietary age supplementation altered gut microbial structure in piglets and growing-finishing pigs

**Figure 6:**
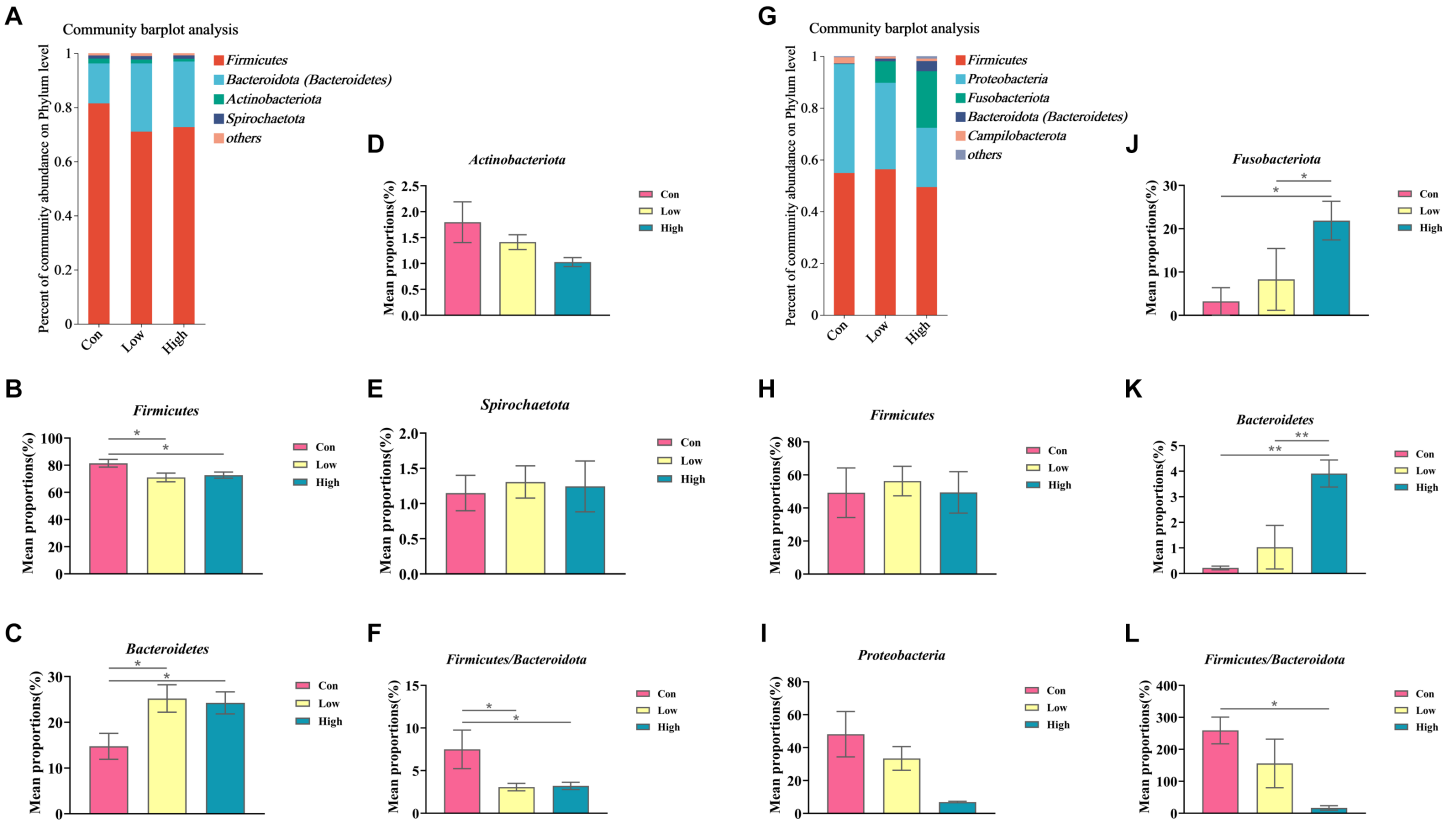
Relative abundance of gut microbiota at the phylum levels. **(A,G)** Phylum-level species composition of piglets and growing-finishing pigs; **(B–F)** Phylum-level species difference analysis of piglets; **(H–L)** Phylum-level species difference analysis of growing-finishing pigs. Data are expressed as the x̄ ± SEM. Statistical significance compared with the control, *p < 0.05 indicates significant differences, whereas **p < 0.01 indicates extremely significant differences.

**Figure 7:**
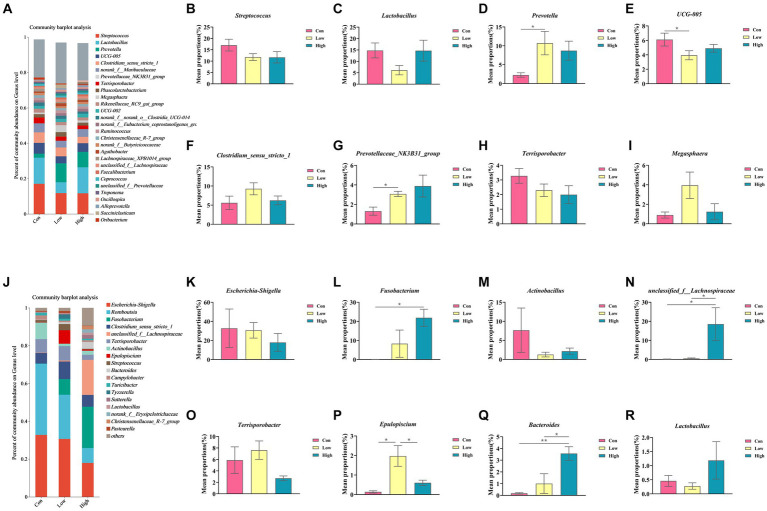
Relative abundance of gut microbiota at the genus levels. **(A,J)** Genus-level species composition of piglets and growing-finishing pigs. **(B–I)** Genus-level species difference analysis of piglets; **(K–R)** Genus-level species difference analysis of growing-finishing pigs. Data are expressed as the x̄ ± SEM. Statistical significance compared with the control, *p < 0.05 indicates significant differences, whereas **p < 0.01 indicates extremely significant differences.

The composition and variation of gut microbiota in piglets and growing-finishing pigs were analyzed at the phylum and genus levels, as shown in Figures 6, 7. Compared with the control group, the linked bar plots of taxon abundance illustrated that low-dose and high-dose groups shifted the relative abundance of bacteria at different taxon levels. During the piglet phase, compared with the control group, the low-dose group increased the relative abundance of *Bacteroidetes* (*p* < 0.05, Figure 6C), *Prevotella* (*p* < 0.05, Figure 7D), and *Prevotellaceae_NK3B31_group* (*p* < 0.05, Figure 7G), while decreasing the relative abundances of *Firmicutes* (*p* < *0.05*, Figure 6B), *Firmicutes* to *Bacteroidetes* (F/B) (*p* < 0.05, Figure 6F), and UCG-005 (*p* < 0.05, Figure 7E). The high-dose group exhibited an increase in the relative abundances of *Bacteroidetes* (*p* < *0.*01, Figure 6C), but a decrease in the relative abundance of *Firmicutes* (*p* < 0.05, Figure 6B) and F/B (*p <* 0.05, Figure 6F).

During the growing-finishing pig phase, the low-dose group increased the relative abundance of *Epulopiscium* (*p* < 0.05, Figure 7P) compared to the control group. The high-dose group increased the relative abundances of *Fusobacteriota* (*p <* 0.05, Figure 6J), *Bacteroidetes* (*p* < 0.01, Figure 6K), *Fusobacterium* (*p* < 0.05, Figure 7L), *unclassified_f__Lachnospiraceae* (*p* < 0.05, Figure 7N), and *Bacteroides* (*p* < 0.01, Figure 7Q), while decreasing the relative abundance of F/B (*p* < 0.05, Figure 6L).

### 3.7 Correlation of dietary age supplementation with serum inflammatory factors and immunoglobulins in association with gut microbiota in piglets and growing-finishing pigs

**Figure 8:**
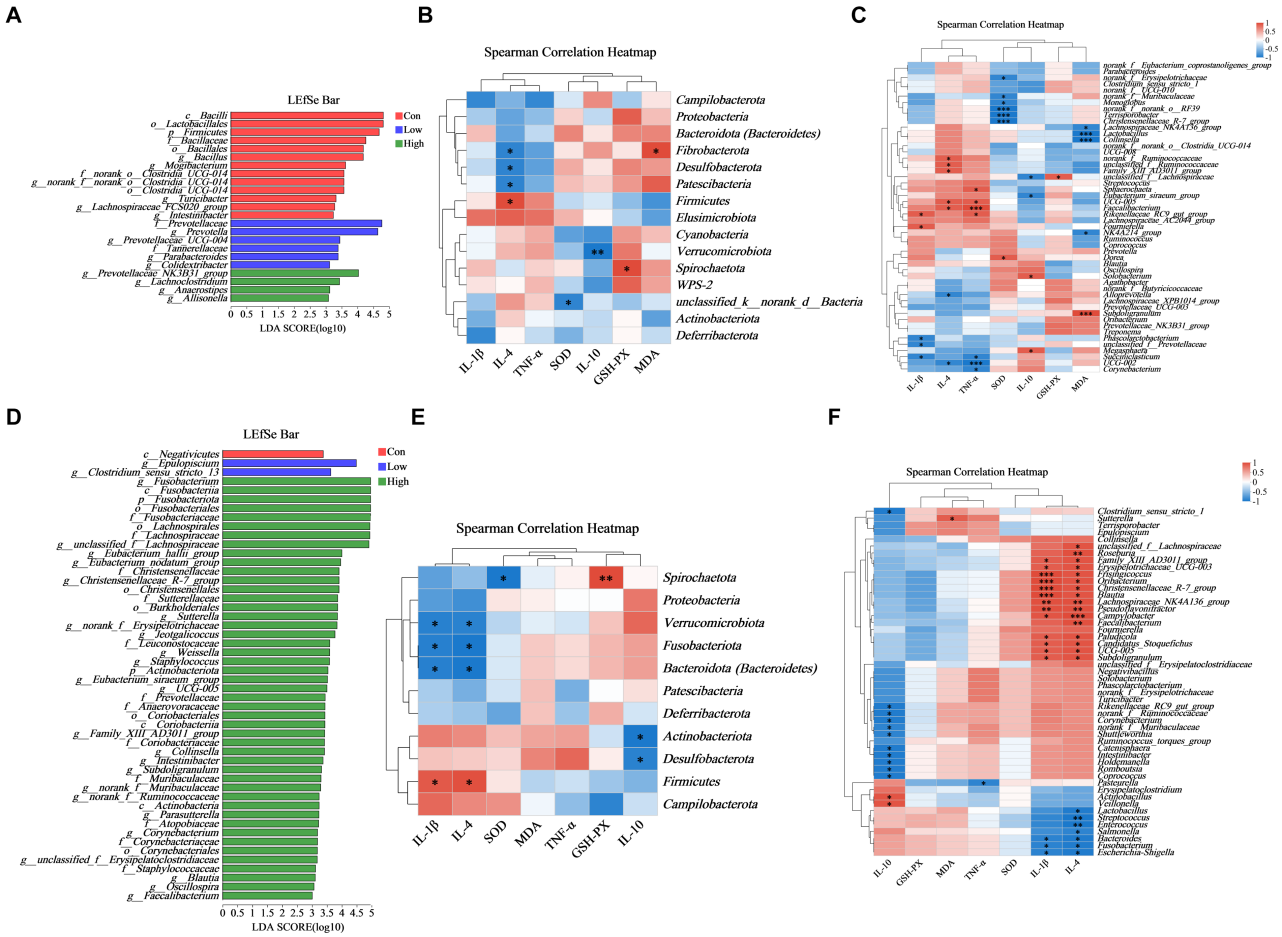
Correlation among phylum and genes in the abundance of key microbial species. **(A,D)** LEfSe analysis identified the phylum to a genus whose abundances significantly differed in each group (LDA score > 3, *p* < 0.05) **(B,C)** Heatmap demonstrates the relationship between serum inflammatory factors, immunoglobulins, and gut microbial diversity at the phylum and genus level in the low-dose group in piglets. For piglet phase, *n* = 6. **(E,F)** Heatmap demonstrates the relationship between serum inflammatory factors, immunoglobulins, and gut microbial diversity at the phylum and genus level in the high-dose group in growing-finishing pigs. For the growing-finishing group, *n* = 5. *p < 0.05 indicates significant differences, whereas **p < 0.01 indicates extremely significant differences.

Based on the LEfSe multistage species difference discrimination analysis, the abundance of *Prevotella* was found to be the highest in the low-dose group during the piglet phase, whereas *unclassified_f_Lachnospiraceae* and *Fusobacterium* exhibited the highest abundance in the high-dose group during the growing-finishing pig phase (Figures 8A,D). Correlation analysis was conducted to assess the relationship between the levels of serum inflammatory and antioxidant factors and the diversity of gut microbiota, as depicted in Figure 8 and Supplementary Figure S1. According to the aforementioned results, we combined the species composition and difference of gut microbiota at the phylum and genus levels in the low-dose group during the piglet phase and the high-dose group during the growing-finishing pig phase, as depicted in Figure 8. By integrating the differential microbiota shown in Figures 6, 7, we identified the *Prevotellaceae_NK3B31_group* as the distinct bacteria negatively correlated with IL-1β, while *Firmicute*s were negatively correlated with IL-10, and *Bacteroidetes* and *Prevotella* were positively correlated with IL-10 in the low-dose group of piglet phase (Figures 8B,C). In the high-dose group of the growing-finishing pig phase, *Bacteroidetes*, *Fusobacteriota*, *Bacteroides*, and *Fusobacterium* were identified as unique bacteria negatively correlated with IL-4 and IL-1β (Figures 8E,F).

### 3.8 Correlation of dietary age supplementation with gram-negative, anaerobic, potentially pathogenic, and biofilm-forming bacteria abundance

**Figure 9:**
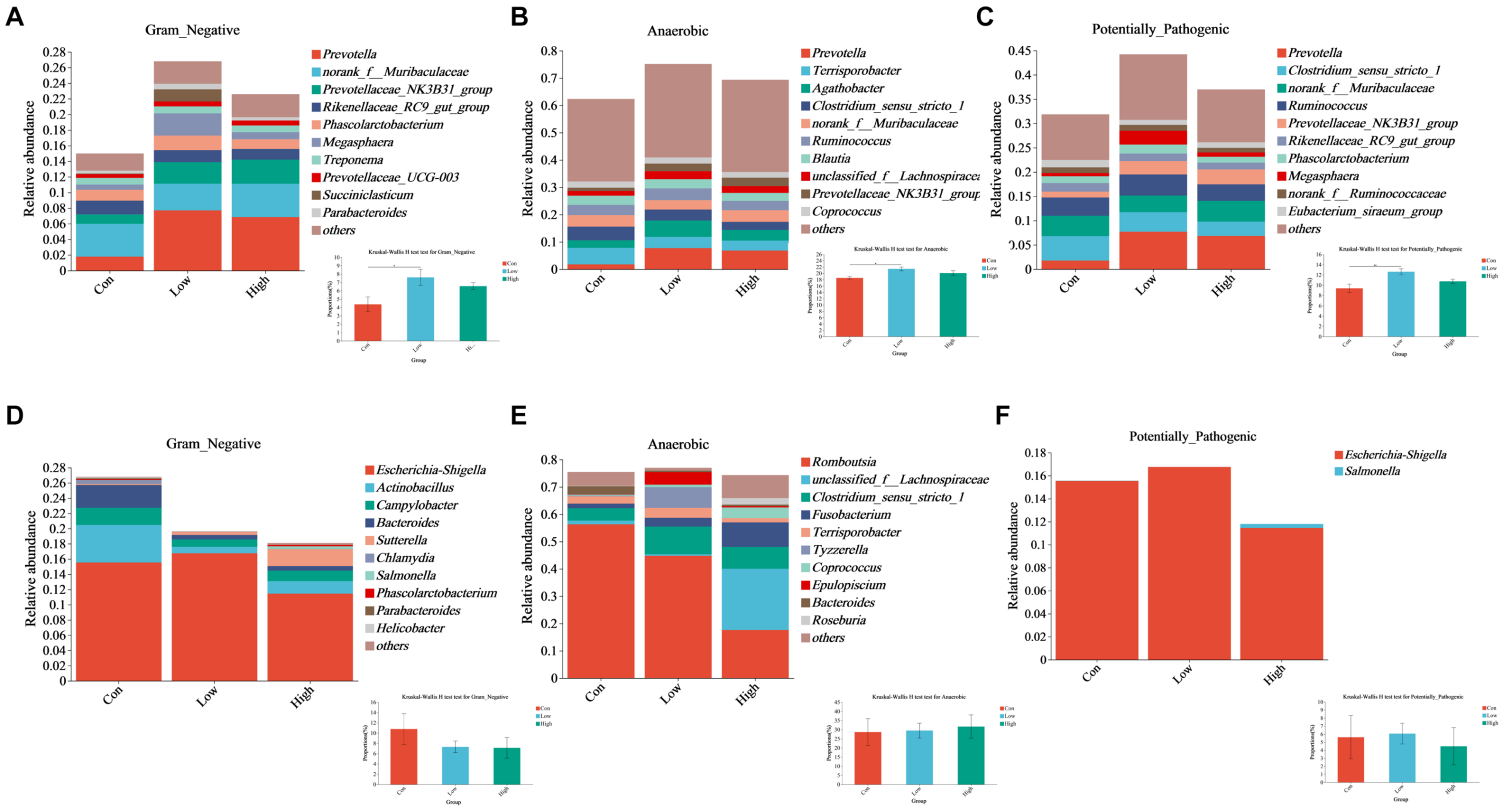
Effects of AGE on the gut microbiota phenotype based on BugBase, including Gram-negative, anaerobic, and potentially pathogenic bacteria. **(A–C)** Relative abundance of Gram-negative, anaerobic, and potentially pathogenic bacteria at the genus level in the piglet phase. **(D–F)** Relative abundance of Gram-negative, anaerobic, and potentially pathogenic bacteria at the genus level in the growing-finishing phase. Each group consists of n = 5. * *p* < 0.05 indicates significant differences, whereas ** *p* < 0.01 indicates extremely significant differences.

Phenotypic analysis and prediction of gut microbiota at the genus level were performed using the BugBase platform. The low-dose group of piglets showed significant differences compared to the control at the genus level in the piglet phase and the growing-finishing phase (*p* < 0.05, Figures 9A–C). The proportions of two genera, *Prevotella* and *Prevotellaceae_NK3B31_group*, increased among the top 10 Gram-negative species in both the low-dose and high-dose groups (Figure 9A). Similarly, *Prevotella*, *Agathobacter*, and *Prevotellaceae_NK3B31_group* genera within the top 10 anaerobic bacterial genera, showed increased proportions in the AGE supplementation groups (Figure 9B). Additionally, *Prevotella*, *Prevotellaceae_NK3B31_group*, and *Megasphaera* (Figure 9C) exhibited increased proportions among the top 10 potentially pathogenic species. However, during the growing-finishing period, the proportions of *Actinobacillus*, *Campylobacter*, and *Bacteroidetes*, three genera within the top 10 Gram-negative species, showed a decrease in both the low-dose and high-dose groups, while *Sutterella* increased (Figure 9D). The proportions of *unclassified_f_Lachnospiraceae*, *Clostridium_sensu_stricto_1*, *Fusobacterium*, and *Coprococcus*, belonging to the top 10 anaerobic species, increased, while *Romboutsia* and *Terrisporobacter* decreased (Figure 9E). Furthermore, the proportion of *Escherichia/Shigella* within potentially pathogenic species decreased in the high-dose group (Figure 9F).

## 4 Discussion

Research has shown that AGE can improve animal growth performance and improve physical health, exhibiting diverse properties including antioxidative capabilities (9, 19), antibacterial activities (20), and anti-inflammatory properties (21). Notably, DMY, myricetin, and quercetin are the principal active ingredients of *Ampelopsis grossedentata*. DMY, in particular, is the most plentiful flavonoid compound with antioxidant and anti-inflammatory activities in *Ampelopsis grossedentata*, primarily targeting the liver and intestines (2). At present, oxidative stress is recognized as a significant factor involving animal health and productivity, with the small intestine being particularly vulnerable to free radical attack. Therefore, maintaining intestinal health is crucial for animal production, and how to prevent and treat intestinal diseases has become a hot topic in the pig industry. In this study, we found that diet supplementation with 500 mg/kg AGE strengthened the health status of the intestinal barrier and improved intestinal morphology in weanling piglets and growing-finishing pigs. Supplementing AGE in the diet can increase the expression of tight junction proteins (such as Zo-1, Claudin-1, and Occludin) to improve the intestinal barrier function of pigs. Additionally, it can regulate the villus height and the V/C ratio of the duodenum, jejunum, and ileum to improve intestinal physiological function. The results of improving intestinal function were validated by the further increase of AGE. Zhang et al. (22) and Qin et al. (23) also found that dietary chlorogenic acid and ellagic acid, both polyphenols and antioxidants, increased the growth properties of piglets by preserving intestinal function.

For weaned piglets, weaning stress induction easily triggers intestinal inflammation, leading to diarrhea and negatively affecting production. However, in this study, we found that adding AGE to the diet prominently increased the level of IL-10 but abated the level of IL-1β in the serum of piglets, which coincided with previous research findings (24). This suggests that AGE may have the potential to inhibit intestinal inflammation. During the piglet phase, immune function and defense capability are particularly weak, making them susceptible to various conditions of oxidative stress and intestinal damage (25). To cope with this stress and damage, antioxidant enzymes play an important role in the body. SOD, CAT, and GSH-PX can eliminate free radicals in the body and are important antioxidant enzymes with protective effects (26). Meanwhile, the content of MDA reflects the degree of tissue oxidative damage and is one of the indicators of oxidative stress intensity (27). Therefore, enhancing antioxidant capacity is crucial for the healthy development of piglets. Vine tea possesses high polyphenol contents, which are significantly positively correlated with antioxidant ability (9). Our research results demonstrated that adding AGE to the diet enhanced its intestinal antioxidant ability, as indicated by increased activity of serum SOD and GSH-Px and reduced content of MDA in the peripheral circulation. This suggests that AGE may be an effective antioxidant that can help piglets cope with oxidative stress and intestinal damage. It is worth noting that this study also found that piglets in the low-dose group (500 mg/kg AGE) had significantly higher serum Ig contents and anti-inflammatory factor expressions compared to the high-dose group (1,000 mg/kg AGE). This suggests that the dosage of AGE can be halved during the growing-finishing period. Interestingly, after 154 days, our findings indicated that the high-dose group (500 mg/kg AGE) exhibited higher antioxidant and anti-inflammatory capacities compared to the low-dose group (250 mg/kg AGE). It indicated that the dosage of 500 mg/kg AGE might be more suitable to be added to the diet.

Furthermore, we also determined the changes in the gut microbiota through 16S rRNA sequencing. The results demonstrated that the high-dose group had more OTUs than the control group in the growing-finishing phase, while no remarkable differences were noticed in the piglet phase. Then, the changes in gut microbiota beta diversity, as presented by PCoA, showed significant differences among all groups in each phase. This further confirms that long-term feeding of 500 mg/kg AGE can improve the composition and richness of the gut microbiota in swine. Similar studies have also observed that other polyphenol-feeding diets can modulate the gut microbiota of pigs (15). For example, the water extract of silver fir (*Abies alba*) is rich in various polyphenols, which have antioxidant and anti-inflammatory effects (28), and is nutritious for some *Lactobacillus* bacteria, and can be used as a prebiotic (29). Cocoa and its products are rich sources of polyphenols, such as flavanols, which can modulate the composition of the gut microbiota by exerting prebiotic mechanisms and enhancing the growth of beneficial gut bacteria, such as *Lactobacillus* and *Bifidobacterium*, while reducing the number of pathogenic ones, such as *Clostridium perfringens* (30). Consistent with these studies, our findings revealed distinct alterations in gut microbiota at the phylum and genus levels in the treatment groups compared to the normal diet group. Specifically, 500 mg/kg AGE increased the relative abundance of profitable bacteria, e.g., *Megasphaera*, *Prevotellaceae_NK3B31_group*, and *Prevotella* in the piglet phase. In the growing-finishing phase, 500 mg/kg AGE also increased the relative proportion of profitable bacteria, e.g., *unclassified_f__Lachnospiraceae*, *Bacteroides*, *Lactobacillus*, and decreased the relative abundances of *Escherichia/Shigella* and *Terrisporobacter*. Moreover, new evidence proves that pigs have approximately 19 “core” bacterial genera, some of which, such as *Lactobacillus*, *Bacteroidetes*, and *Fusobacteria*, were conducive to improving the growth properties of pigs (31). Consistent with these studies, further evidence of a clear connection between gut microbiota and inflammatory cytokines has been provided through correlation analysis. Combined with the significantly changed microbial species at the phylum and genus levels, we speculate that AGE can improve the intestinal barrier and reduce the inflammatory response, which is related to the composition of the gut microbiota, as shown by the negative correlation observed between the increase of *Prevotellaceae_NK3B31_group* and proinflammatory factor IL-1β, as well as the positive correlation between the increase of *Prevotella* and IL-10 in the piglet phase of the low-dose group (500 mg/kg AGE). It has been reported that *Prevotellaceae* and *Lachnospiraceae* contribute to piglets absorbing energy from plant-derived feed (32, 33). There was no significantly different microbiota in the association analysis of growing-finishing pigs under different conditions initially; however, upon a separate association analysis focusing on the high-dose group (500 mg/kg AGE) of growing-finishing pigs, a remarkable increase in the genus *Bacteroidetes* was discovered. Notably, this genus had a negative correlation with proinflammatory factors. Previous research has highlighted the advantageous effects of *Bacteroidetes* on the host, as they can prevent colonization and infection by potential pathogens in the intestine (34, 35). In addition, the correlation analysis found that the genus *Firmicutes* was positively correlated with proinflammatory factors, as studies showed that the ratio of F/B was an important marker of gut inflammation (36). It was found that polyphenols and polysaccharides played an important role in regulating gut microflora (the phyla *Bacteroidetes*, *Firmicutes,* and *Firmicutes*/*Bacteroidetes* ratio) through signal transduction of short-chain fatty acids, bile acids, and lipopolysaccharides and played an anti-inflammatory role (12, 37). This may also be the reason for a significant decrease in the ratio of F/B in the high-dose group (500 mg/kg AGE).

Given the differences in gut microbiota between piglets and growing-finishing pigs, we used BugBase for prediction to better understand the potential phenotypes and functional predictions of gut microbiota in response to distinct environments (38). We found a visible decrease in the high proportion of latently pathogenic bacteria and Gram-negative microflora in the high-dose group (500 mg/kg AGE) in the course of the growing-finishing phase of pigs. This reduction was mainly attributed to changes in the abundance of *Escherichia/Shigella* and *Actinobacillus*. It is worth noting that *Escherichia/Shigella* is closely associated with intestinal inflammation, and previous studies have shown that they may contribute to the differentiation of the gut microbiota of colorectal cancer patients and healthy individuals (39, 40). Moreover, *Actinobacillus* species are Gram-negative bacteria known to cause various animal diseases, including pleuropneumonia, endometritis, diarrhea, and intestinal inflammation (41–44). In contrast, during the piglet period, we found that the dietary AGE significantly increased the abundance of anaerobic microbials in the low-dose group (500 mg/kg AGE), mainly attributed to the altered abundance of *Prevotella*. The gut microbiota is a complex community, with the highest abundance being *Firmicutes* (Gram-positive bacteria) and *Bacteroidetes* (Gram-negative bacteria), which are highly sensitive to oxygen. Therefore, the gut ecosystem is essentially anaerobic (45). *Prevotella*, a Gram-negative anaerobic bacteria belonging to the *Bacteroidetes* phylum, plays a vital role in the digestion of dietary fiber and is considered to have probiotic functions in humans (46). According to numerous studies, *Prevotella* spp. is one of the most plentiful bacteria in the gastrointestinal tract of pigs aged 28 to 91 days (47–49). Therefore, it can be inferred that a dosage of 500 mg/kg AGE can improve the anaerobic traits of the piglet’s intestine and reduce the presence of pathogenic bacteria in the intestines of growing-finishing pigs. In conclusion, another potential mechanism through which AGE mitigates intestinal inflammation in pigs is by reducing the population of harmful bacteria in the intestines.

## 5 Conclusion

Based on all the results, we have demonstrated the positive effects of incorporating AGE into diets on the growth properties, intestinal damage, intestinal capacity of antioxidants, and gut microbiota diversity of pigs at different stages. Our findings revealed that dietary supplementation of 500 mg/kg can effectively modulate the microbial composition, suppress inflammatory factors, and enhance the intestinal antioxidant ability of pigs. These results highlight the beneficial influences of AGE in facilitating the weaning transition of piglets and promoting the healthy growth of growing-finishing pigs.

## Data availability statement

The original contributions presented in the study are publicly available. This data can be found here: NCBI BioProject, accession PRJNA1131186.

## Ethics statement

The animal study was approved by The Animal Care and Use Committee of Hunan Agricultural University. The study was conducted in accordance with the local legislation and institutional requirements.

## Author contributions

XL: Methodology, Writing – original draft. FZ: Writing – original draft, Data curation, Formal analysis. ML: Investigation, Visualization, Writing – original draft. RL: Software, Writing – review & editing. ZZ: Data curation, Writing – review & editing. JX: Methodology, Validation, Writing – review & editing. LW: Funding acquisition, Writing – review & editing. RfL: Conceptualization, Writing – review & editing.

## Footnotes

^1^ www.isanger.com
