# Supplementary material for: Supplementation of Ampelopsis grossedentata extract contributes to the improvement of intestinal health in swine
Source: Front Vet Sci. 2024 Aug 21;11:1417309. doi: 10.3389/fvets.2024.1417309 (PMC11373254; doi:10.3389/fvets.2024.1417309)
Supplement: Supplementary file 1 [file Table_1.DOCX]

| Gene | Primer sequences (5^，^-3^,^)^a^ | Gene ID and Product length |
| --- | --- | --- |
| *IL-10* | F: TAGGGTGTGCCCTATGGTGT | ID:397106 (114bp) |
|  | R: GGGTGGGTAGGCTTGGAATG |  |
| *IL-1β* | F: GTGGCAGGACCTACACTCTTC | ID:396565 (115bp) |
|  | R: TTCCTTCAGAATGCCGTCCT |  |
| *Zo-1*  *Occludin*  *Claudin-1*  *Actin* | F: AAGGTCTGCCGAGACAACAG  R: TCACAGTGTGGTAAGCGCAG  F: TCAGGTGCACCCTCCAGATT  R: ATGGGCGTCTCTCCACCATA  F: GGACAAAACCGTGTGGGAAC  R: ACATGAAAATGGCTTCCCTCC  F: GCAATCAGGGGATGAGGACA  R: CAATGGACGGGAAAACAGCC | ID:100736682 (137bp)  ID:397236 (189bp)  ID:100625166 (200bp)  ID:733615 (196bp) |

**Table S1 Primer sequences used for quantitative real-time PCR**

An F means forward, and an R means reverse.
